# PreDisorder: ab initio sequence-based prediction of protein disordered regions

**DOI:** 10.1186/1471-2105-10-436

**Published:** 2009-12-21

**Authors:** Xin Deng, Jesse Eickholt, Jianlin Cheng

**Affiliations:** 1 Department of Computer Science, University of Missouri-Columbia, Columbia, MO 65211, USA; 2 Informatics Institute, University of Missouri-Columbia, Columbia, MO 65211, USA; 3 C Bond Life Science Center, University of Missouri-Columbia, Columbia, MO 65211, USA

## Abstract

**Background:**

Disordered regions are segments of the protein chain which do not adopt stable structures. Such segments are often of interest because they have a close relationship with protein expression and functionality. As such, protein disorder prediction is important for protein structure prediction, structure determination and function annotation.

**Results:**

This paper presents our protein disorder prediction server, PreDisorder. It is based on our *ab initio *prediction method (MULTICOM-CMFR) which, along with our meta (or consensus) prediction method (MULTICOM), was recently ranked among the top disorder predictors in the eighth edition of the Critical Assessment of Techniques for Protein Structure Prediction (CASP8). We systematically benchmarked PreDisorder along with 26 other protein disorder predictors on the CASP8 data set and assessed its accuracy using a number of measures. The results show that it compared favourably with other *ab initio *methods and its performance is comparable to that of the best meta and clustering methods.

**Conclusion:**

PreDisorder is a fast and reliable server which can be used to predict protein disordered regions on genomic scale. It is available at http://casp.rnet.missouri.edu/predisorder.html.

## Background

While most regions of a protein adopt localized, stable structures, there are some segments of the protein chain which do not. These are regions whose coordinates are hard to determine by experimental techniques or that simply do not fold into stable structures [1,2]. Such regions are known as disordered regions. Proteins with disordered regions are capable of binding to multiple partners and participating in various reactions and pathways [3-5]. Disordered regions can also give rise to the poor expression of a protein, making it difficult to produce for crystallization or other purposes [6]. Consequently, the prediction of disordered regions in proteins has implications for protein production, structure prediction and determination, function annotation and cellular process recognition.

Measuring native disorder experimentally is time consuming and expensive and thus computational approaches for the prediction of protein disordered regions have received considerable attention in recent years [7]. As a result, a number of disorder prediction software and web services and their underlying methods are quickly becoming a valuable tool for protein structure prediction, determination, and function annotation [8-18]. To stimulate further development of disorder prediction, CASP has dedicated a category to blindly benchmark the current state of the art. Here we benchmark our *ab initio *and consensus (or meta) disorder predictors along with dozens of other predictors that participated in the CASP8 experiment. The good performance of our PreDisorder server makes it a valuable and accurate tool for protein structure prediction, protein determination and protein engineering.

## Implementation

### Ab initio neural network method

Our server, PreDisorder, is based on our *ab initio *method that participated in CASP8 under the group name MULTICOM_CMFR. This is a machine learning approach using 1-D Recursive Neural Networks. With this approach, a target protein sequence is first aligned against several template profiles using PSI-BLAST. This creates an input profile of the sequence. This profile along with the predicted secondary structure and solvent accessibility is fed into a 1D Recursive Neural Network (1D-RNN) that makes the disorder predictions [6]. More specifically for each protein sequence, the input is a 1-dimentional array *I *whose length is the total number of the residues in the sequence. Each element *I*_*i *_of the array is a vector with 25 values which represent the residue *i*. Of these 25 values, 20 represent the frequencies of each amino acid at the corresponding position from PSI-BLAST profile [19]. The other five are binary values used to encode the predicted secondary structure (Helix, Strand or Coil) and solvent accessibility of the residue [20-22]. Based on the input *I*, the 1D-RNN produces an array of real numbers *O*, where the *i*^*th *^element *O*_*i *_is the probability that the *i*^*th *^residue will be disordered. A large curated dataset was randomly divided into ten subsets of approximately equal size in the preparation for the following ten-fold cross-validated training and testing. And then, this 1D-RNN was trained and cross-validated using the ten subsets [23]. Finally, the predicted disorder probabilities of the residues were re-scaled so that the ratio of residues with disorder probability greater than or equal to 0.5 is close to the ratio of the disorder residues in the training dataset [23]. Specifically, the scaling method first identified a probability threshold *t (e.g. 0.1) *for selecting predicted disorder residues such that the ratio (the number of predicted disordered residues/the number of total residues in the test dataset) is equal to the ratio of disorder residues in the training dataset (e.g. 5%). And then the predicted disorder probabilities (*x*) was re-scaled as *x*/t * 0.5 (if *x *<= *t*) or 0.5 + 0.5 * (*x *- *t*)/(*1 *- *t*) (if *x *>*t*).

### Meta method

A meta method is a consensus approach that makes predictions based on the output of other predictors. Similar ideas have been applied to solve many prediction problems such as protein fold recognition and achieved much better performance than individual predictors. One such example of this approach is 3D-Jury. 3D-Jury is an automated protein structure meta prediction system available through Meta Server, and it generates meta-predictions from a variety of models gained by variable methods [24][25][26]. Our new meta predictor MULTICOM makes predictions based on a consensus formed from other CASP8 disorder predictors. It removes a few very inaccurate disorder predictors and then averages the output of the remaining disorder predictors. Our simple averaging approach is different from other meta methods based on consensus voting.

## Results and discussion

We evaluated 27 disorder predictors that participated in CASP8. Among these predictors were our *ab initio *method predictor (MULTICOM-CMFR) and meta predictor (MULTICOM). They were evaluated on 117 protein targets whose structures were available when our evaluation was conducted. These targets contain 25431 residues and all the disorder predictions for them were downloaded from the CASP8 web site [27]. When evaluating the disorder predictions against the protein targets, target residues that did not have corresponding coordinates in its PDB file were considered to be disordered. The disorder annotations for the targets were curated by Dr.McGuffin [28]. Each residue in the target sequence is tagged with a binary label of "O" (order) or "D" (disorder). We evaluated the methods on all 117 targets and two subsets (97 X-ray structures and 20 NMR structures), respectively. It is worth pointing out that our evaluation serves as a complementary, comparative benchmark of our methods. Readers should refer to the CASP8 assessment paper for the official assessment of disorder predictions [29].

In evaluating the disorder predictors, we considered a number of different, commonly used measurements of performance for binary classifiers. One such measurement was the ROC score. This value represents the area under the Receiver Operating Characteristic (AUC) curve and measures the performance of a classifier system and its dependence upon its discrimination threshold. Ranking the predictors using ROC curves is a widely used method in bioinformatics and CASP competitions [7,30,31].

Another set of commonly used measurements for classifier systems are sensitivity and specificity. For each disorder predictor, we calculated the Positive Sensitivity (), Positive Specificity (),

Negative Sensitivity () and Negative Specificity () [31]. Here, TP is the number of true positives (residues correctly identified as disordered) and FP is the number of false positives (residues predicted as disordered, but experimentally ordered). TN is the number of true negatives (residues correctly identified as ordered) and FN the number of false negatives (residues predicted as ordered, but experimentally disordered).

While in principle it is possible for a system to achieve both high values for positive and negative sensitivity, in practice it does not happen often. Usually, a sharp increased in one, results in a decrease in the other. An extreme example would be a predictor which identifies all residues as disordered. Such a system would have a positive sensitivity of 100% and a negative sensitivity of 0%. In an attempt to join several of these measurements into one, we considered the product of positive sensitivity and negative sensitivity and the harmonic mean, or F-measure, of the positive sensitivity and positive specificity [32].

We also calculated a weighted score for each predictor. This is a measure which was introduced in CASP6 and is defined as Score () where W_disorder _is set to 92.63 and W_order _to 7.37 [31]. As defined, this measure greatly rewards disordered residues correctly identified as classified as disordered while heavily penalizing any disordered residue that is misclassified.

**Figure 1:**
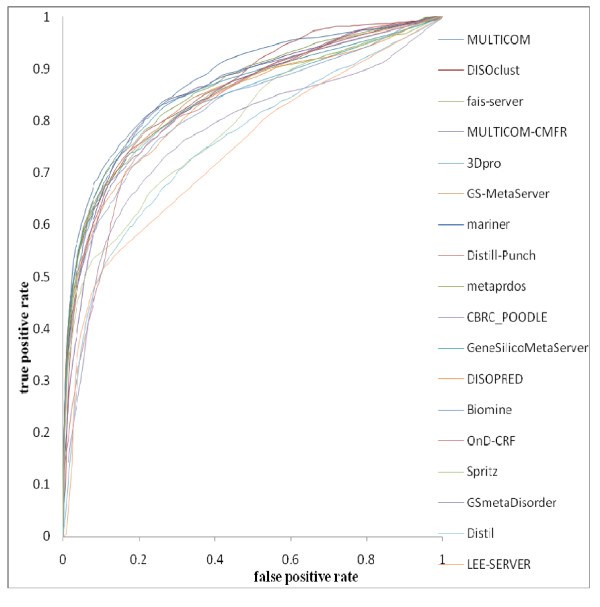
**ROC curves of CASP8 predictors (ordered by ROC score) on the CASP8 dataset which consisted of 117 protein targets**.

Table 1 reports the ROC scores, weighted score, positive sensitivity, negative specificity, negative sensitivity, negative specificity, product of positive sensitivity and negative sensitivity, F-measure respectively of all the disorder predictors. Moreover, Table 1 also shows the total number of residues predicted by each predictor respectively. For comparison, we also repeated the evaluation for the "only x-ray" and the "only NMR" sets, and the results are shown in Table 2 and Table 3. Figure 1 shows the ROC curves for the predictors. The predictors are ordered by ROC scores since the ROC measure is probably the most balanced measurement.

**Table 1:** Results for protein disorder predictors that participated in CASP8 on 117 targets.

Disorder Predictor	ROC Score	Weighed Score	Total Res.	Pos. Sens.	Pos. Spec.	Neg. Sens.	Neg. Spec.	Sens. Prod.	F-meas.
MULTICOM	0.879	6.89	25148	0.532	0.619	0.973	0.962	0.518	0.572
DISOclust	0.862	7.822	24021	0.753	0.248	0.813	0.976	0.612	0.373
fais-server	0.861	6.613	24021	0.522	0.533	0.963	0.961	0.503	0.528
MULTICOM-CMFR*	0.859	8.06	25431	0.724	0.299	0.859	0.974	0.622	0.423
3Dpro*	0.855	4.826	23934	0.378	0.727	0.988	0.949	0.373	0.497
GS-MetaServer	0.852	5.356	24976	0.41	0.711	0.986	0.953	0.404	0.52
mariner1*	0.846	7.469	23148	0.622	0.396	0.923	0.968	0.574	0.484
Distill-Punch*	0.846	0.798	23311	0.067	0.75	0.998	0.93	0.067	0.123
Metaprdos	0.842	6.431	22363	0.505	0.539	0.966	0.961	0.488	0.522
Distill-Punch*	0.839	0.823	23730	0.063	0.881	0.999	0.93	0.063	0.118
CBRC_POODLE*	0.839	6.259	24021	0.522	0.403	0.937	0.96	0.489	0.455
GeneSilicoMeta	0.839	6.338	24976	0.489	0.627	0.976	0.959	0.478	0.55
DISOPRED*	0.831	6.497	24021	0.522	0.485	0.955	0.961	0.498	0.503
Biomine*	0.825	6.394	23472	0.517	0.47	0.952	0.96	0.492	0.493
OnD-CRF*	0.82	5.214	24021	0.47	0.31	0.914	0.955	0.429	0.373
Spritz*	0.791	5.431	24021	0.434	0.514	0.966	0.954	0.42	0.471
GSMetaDisorder	0.772	6.31	24504	0.579	0.289	0.88	0.961	0.51	0.386
Distill*	0.758	5.637	24021	0.671	0.173	0.738	0.965	0.495	0.276
LEE-SERVER*	0.741	3.595	21955	0.31	0.449	0.968	0.943	0.3	0.367

Results of our evaluation of all the protein disorder predictors ranked by ROC Score (AUC). Total Res. represents the total number of residues predicted. Pos. Spec. and Neg. Spec. are the positive and negative specificities. Pos. Sens. and Neg. Sens. are the positive and negative sensitivities. F-meas. is the F-Measure of the positive sensitivity and positive specificity and Sens. Prod. is the product of the positive sensitivity and negative sensitivity. * denotes *ab initio *methods. MULTICOM-CMFR is the predictor name of PreDisorder in CASP8.

**Table 2:** Results for protein disorder predictors that participated in CASP8 on the 20 NMR targets (T0437, T0460, T0462, T0464, T0466, T0467, T0468, T0469, T0471, T0472, T0473, T0474, T0475, T0476, T0480, T0482, T0484, T0492, T0498, T0499).

Disorder Predictor	ROC Score	Weighed Score	Total Res.	Sens.	Spec.	Sens. Prod.	F-measure.
GSMetaDisorder	0.800	3.801	1654	0.786	0.700	0.550	0.082
MULTICOM-CMFR*	0.792	4.393	1895	0.816	0.700	0.572	0.125
fais-server	0.769	5.407	1601	0.510	0.874	0.446	0.186
OnD-CRF*	0.761	5.671	1601	0.429	0.925	0.397	0.226
MULTICOM	0.760	5.283	1895	0.469	0.878	0.412	0.155
Biomine*	0.753	4.753	1489	0.465	0.845	0.393	0.139
CBRC_POODLE*	0.744	3.864	1601	0.490	0.774	0.379	0.113
DISOPRED*	0.741	5.458	1601	0.531	0.870	0.462	0.188
Metaprdos	0.731	5.971	1601	0.449	0.938	0.421	0.263
3Dpro*	0.729	6.115	1411	0.458	0.948	0.434	0.312
Distill*	0.720	3.042	1601	0.694	0.636	0.441	0.105
GS-MetaServer	0.715	5.525	1775	0.367	0.933	0.343	0.197
DISOclust	0.709	3.011	1601	0.571	0.682	0.390	0.098
Distill-Punch*	0.703	4.107	1601	0.000	0.986	0.000	0.000
GeneSilicoMeta	0.703	5.540	1775	0.469	0.897	0.421	0.185
Spritz*	0.679	4.772	1601	0.224	0.943	0.212	0.149
Mariner1*	0.666	4.636	1601	0.592	0.788	0.466	0.143
LEE-SERVER*	0.654	5.486	1684	0.349	0.932	0.325	0.176

* denotes *ab initio *methods.

**Table 3:** Results for protein disorder predictors that participated in CASP8 on the 97 x-ray targets.

Disorder Predictor	ROC Score	Weighed Score	Total Res.	Sens.	Spec.	Sens. Prod.	F-measure.
MULTICOM	0.887	7.021	23253	0.534	0.981	0.523	0.610
DISOclust	0.868	8.165	22420	0.758	0.823	0.624	0.397
fais-server	0.867	6.699	22420	0.523	0.969	0.506	0.555
MULTICOM-CMFR*	0.865	8.355	23536	0.721	0.873	0.630	0.455
3Dpro*	0.862	4.745	22523	0.375	0.990	0.372	0.507
GS-MetaServer	0.861	5.343	23201	0.411	0.991	0.407	0.541
Mariner1*	0.857	7.679	21547	0.623	0.933	0.582	0.518
Distill-Punch*	0.856	0.554	21710	0.069	0.999	0.069	0.128
CBRC_POODLE*	0.848	6.430	22420	0.523	0.949	0.496	0.493
GeneSilicoMeta	0.848	6.399	23201	0.490	0.982	0.481	0.579
Metaprdos	0.846	6.467	20762	0.507	0.968	0.491	0.536
DISOPRED*	0.837	6.571	22420	0.521	0.961	0.501	0.528
Biomine*	0.829	6.506	21983	0.519	0.959	0.498	0.522
OnD-CRF*	0.822	5.181	22420	0.471	0.913	0.430	0.379
Spritz*	0.800	5.478	22420	0.440	0.968	0.426	0.486
GSMetaDisorder	0.778	6.492	22850	0.576	0.894	0.515	0.417
Distill*	0.761	5.822	22420	0.670	0.746	0.499	0.289
LEE-SERVER*	0.749	3.438	20271	0.309	0.971	0.300	0.379

* denotes *ab initio *methods.

The CASP8 disorder prediction methods can be classified into four main categories [33]: (1) Meta method. Predictors like MULTICOM, GS-MetaServer, Metaprdos, GeneSilico, GSMetaDisorder and Distill use this method to fulfill disorder prediction. (2) Clustering method. For instance, it is used by predictor DISOclust. DISOclust first gains multiple 3D models from the nFOLD3 server and then makes disorder predictions by combining the results obtained from running the DISOclust method and DISOPRED3 method. (3) *Ab initio *method. A large number of predictors in CASP8 adopt this method and examples include 3Dpro, Mariner, Spritz, biomine, CBRC_poodle, disopred, OnD-CRF and our predictor MULTICOM_CMFR. (4) Hybrid method. Fais-server is a hybrid method that combines both *ab initio *predictions and homology-based template information. Both *ab initio *and hybrid methods usually exist as standalone packages, while meta methods rely on other predictors.

In examining the results, no one method appears to perform decisively better than the rest according to all the measures. Predictors from each of the three types of methods (*ab initio*, meta and clustering) are represented in the top seven when comparing the predictors only on the basis of ROC score, weighted score, specificity or sensitivity. The meta method MULITCOM, the clustering method DISOclust, the hybrid method Fais-server and *ab initio *method MULTICOM-CMFR and 3Dpro are among top 5 in terms of ROC scores. Other *ab initio *predictors such as mariner1 and Distill-Punch also performed well. Interestingly, our *ab initio *predictor MULTICOM-CMFR also ranks first in weighted score and product of positive and negative sensitivity. Being an *ab initio *method, it also has the benefit of being able to make predictions solely on an input sequence. The other types of methods need additional information such as output from other predictors (e.g. meta methods), tertiary structure models (clustering methods), or homologous structure templates (hybrid methods). Consequently, our PreDisorder server based on MULTICOM-CMFR is generally an accurate predictor that can be applied to the genome-scale annotation of protein disordered regions. Especially regarding the limits of predictability of intrinsically disordered residues from crystallographic experiments, both of our methods performed well on the X-ray targets shown in Table 3[34]. Several methods (e.g., MULTICOM, DISOclust, fais-server, MULTICOM-CMFR, 3Dpro, mariner and Distill-Punch) yield similarly good AUC scores (>= 0.846), suggesting that the accuracy of disorder predictions might be close to the limit [34].

All of the predictors do quite well with respect to negative specificity and negative sensitivity. This is not too surprising as the most of the residues in a protein are ordered and hence the number of true negatives (TN) is very close to the true negatives plus false positives (TN+FP) and to the true negatives plus the false negatives (TN+FN).

## Conclusion

This paper presents our disorder prediction web server, PreDisorder, and evaluates its performance against several other disorder predictors. We benchmarked MULTICOM-CMFR, the method employed by Predisorder and our meta method MULTICOM, along with several other protein disorder predictors on the 117 targets used in CASP8. The results show that our method is among the best and provides reliable protein disordered region predictions. Therefore, our server (PreDisorder) is a useful tool for structural and functional genomics.

## Availability and Requirements

Project name: PreDisorder

Project Home Page: http://casp.rnet.missouri.edu/predisorder.html

Operating system(s): Platform independent (web server)

Programming languages: Perl, C, C++

Other requirements: None

License: Web application is freely accessible for all users.

Any restrictions to use by non-academics: None

**Figure 2:**
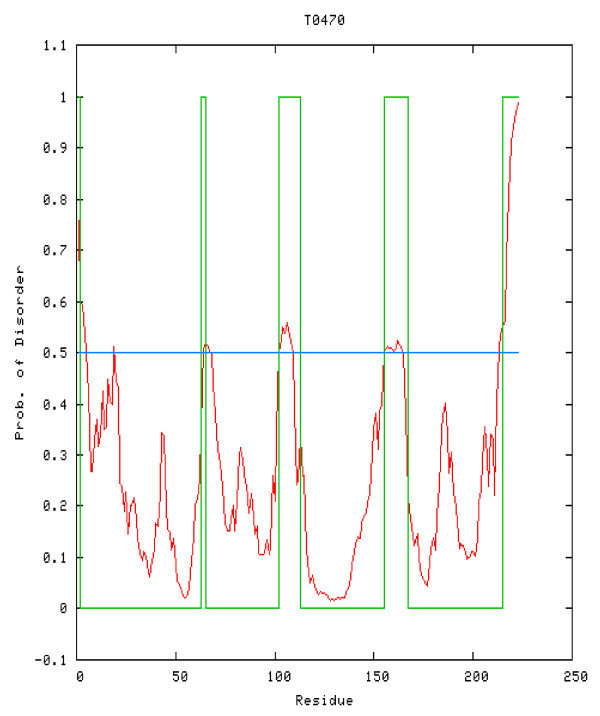
**Example output from PreDisorder showing probability of disorder for each residue in a sequence (CASP8 target T0470)**. The red curve represents predicted disorder probabilities. The green curve denotes real disorder annotations (1 - disorder; 0 - not disorder).

The use of PreDisorder is straight forward and takes place through a simple input form. The input form requires only three inputs: email address, target name and protein sequence. PreDisorder can make predictions in a very short time and sends the results back to users via email. Disorder prediction results include the user-defined target name, the author, any predictor remarks and the disorder predictions. These predictions are in CASP format and occupy several lines. Each line contains the residue code, an order or disorder assignment code and the number specifying the associated probability of disorder. We also return the results in graphical form, as seen in Figure 2. In this graph, users can visualize changes in the likelihood of disorder from residue to residue over the submitted sequence. The red curve shows our predicted probability of disorder for each residue in the target sequence, the green curve represents the determined disorder result by biological experiment for the target. In addition, the blue line y = 0.5 represents the threshold we chose to judge the probability of disorder for a residue.

## Authors' contributions

JC designed and implemented the disorder prediction methods and conducted CASP8 experiments. XD evaluated the predictors. XD and JE wrote the first draft of the manuscript. DX, JE and JC set up the web server. All the authors edited the manuscript and approved it.
